# Bailout using NBCA for incomplete onyx embolization of tentorial dural arteriovenous fistula

**DOI:** 10.1016/j.radcr.2024.07.138

**Published:** 2024-08-22

**Authors:** Masahiro Sugihara, Atsushi Fujita, Takeshi Kondoh, Yoshiyuki Takaishi, Hirotomo Tanaka, Nao Tachizawa, Takashi Sasayama

**Affiliations:** a Department of Neurosurgery, Shinsuma General Hospital, 3-1-14 Kinugake-cho, Suma-ku, Kobe 654-0048, Japan; b Department of Neurosurgery, Kobe University Graduate School of Medicine, 7-5-1 Kusunoki-cho, Chuo-ku, Kobe 650-0017, Japan

**Keywords:** Dural arteriovenous fistula, Transarterial embolization, Onyx, NBCA

## Abstract

Transarterial embolization using Onyx (Medtronic, Irvine, CA, USA) results in a high cure rate for complete obliteration of dural arteriovenous fistulas. However, incomplete obliteration occurs in some cases. Reports on the use of bailout therapy in such cases are limited. A 79-year-old man was diagnosed with Borden type III tentorial dural arteriovenous fistulas during a check-up for a headache. We first performed transarterial embolization with Onyx from a tentorial artery, but the fistula was not completely obliterated. We then performed an additional transarterial embolization with n-butyl-2-cyanoacrylate from the same artery in a single session, and the fistula was successfully bailed out, resulting in complete obliteration. Combining different liquid embolic materials, Onyx and n-butyl-2-cyanoacrylate, is an effective strategy for achieving complete obliteration in incomplete transarterial embolization treatment of dural arteriovenous fistulas.

## Introduction

Transarterial embolization (TAE) with Onyx (Medtronic, Irvine, CA, USA) has been reported to result in a high cure rate for the complete occlusion of dural arteriovenous fistulas (DAVFs). However, some cases result in incomplete occlusion [[1], [2], [3], [4]], and there are limited reports on effective bailout strategies for these situations. This report presents a unique case of a tentorial DAVF (TDAVF) where initial TAE with ONYX resulted in incomplete occlusion. This case is particularly noteworthy as it demonstrates a successful bailout method using a combination of Onyx and n-butyl-2-cyanoacrylate (NBCA) to achieve complete occlusion. Typically, incomplete occlusion from one feeder prompts consideration of embolism from another feeder or evaluation of alternative treatments such as open surgery or Gamma Knife therapy. In this case, the high navigability of modern microcatheters facilitated reaccess to narrow feeders already filled with embolic material. The combined use of Onyx for initial embolization and NBCA for additional embolization leveraged the different properties of these liquid embolic agents. This case highlights the potential for combining different embolic agents to overcome their limitations, providing valuable insights for clinicians facing similar challenges.

## Case description

**Fig. 1:**
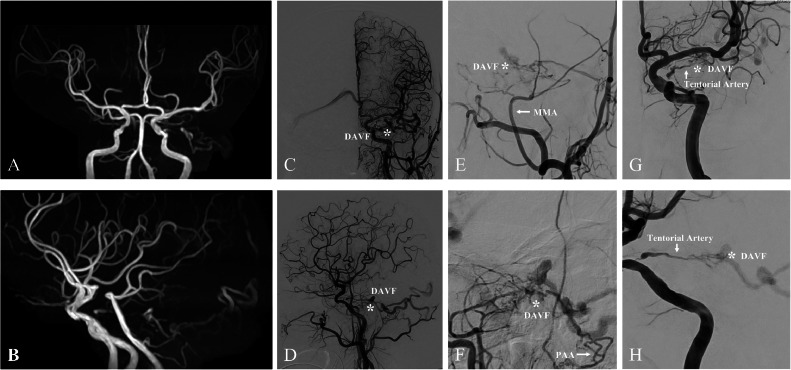
The shunt point of DAVF is indicated (asterisk). (A,B) Magnetic resonance imaging (MRI) reveals a left tentorial DAVF. (C,D) Digital subtraction angiography (DSA) of the left common carotid artery showing that the tentorial DAVF (asterisk) is fed from the left tentorial, left middle meningeal, and left posterior auricular arteries. A shunt pouch is formed in the left petrotentorial location. Drainage occurs via arterialization of the left petrosal vein, forms a varix, and drains into the confluence of the right transverse sinus. (E,F) DSA of the left external carotid artery showing that the middle meningeal artery (MMA) and posterior auricular artery (PAA) are tortuous and inaccessible near the shunt point of DAVF (asterisk). (G,H) DSA of the left internal carotid artery showing that the tentorial artery is a relatively straight and well-dilated vessel to the DAVF (asterisk), but the origin of the meningohypophyseal trunk (MHT) is highly stenotic.

**Fig. 2:**
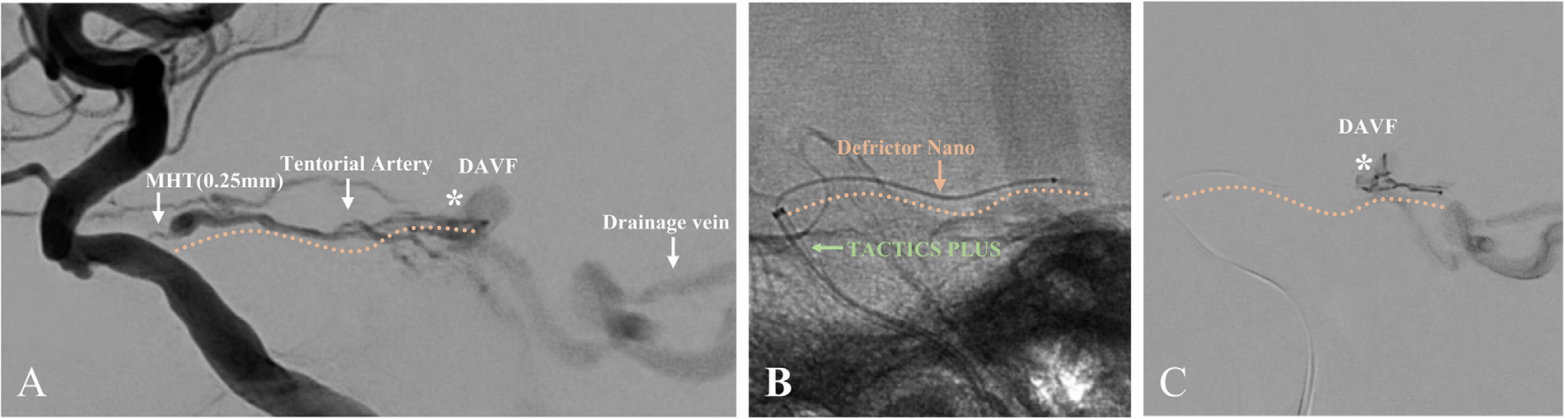
First embolization using Onyx: yellow line indicates the position of the microcatheter. (A) DSA of the left internal carotid artery showing that the origin of MHT is narrowed to 0.25 mm. The DeFrictor Nano tip diameter is 0.43 mm, and the length of the thin part is 6 cm, which is thicker than the diameter of the origin of MHT. (B) The TACTICS PLUS (green arrow) is positioned immediately adjacent to the origin of the MHT to obtain backup support when passing through the stenotic lesion. (C) The DeFrictor Nano catheter easily passes through the stenotic lesion and reaches near the DAVF (asterisk).

**Fig. 3:**
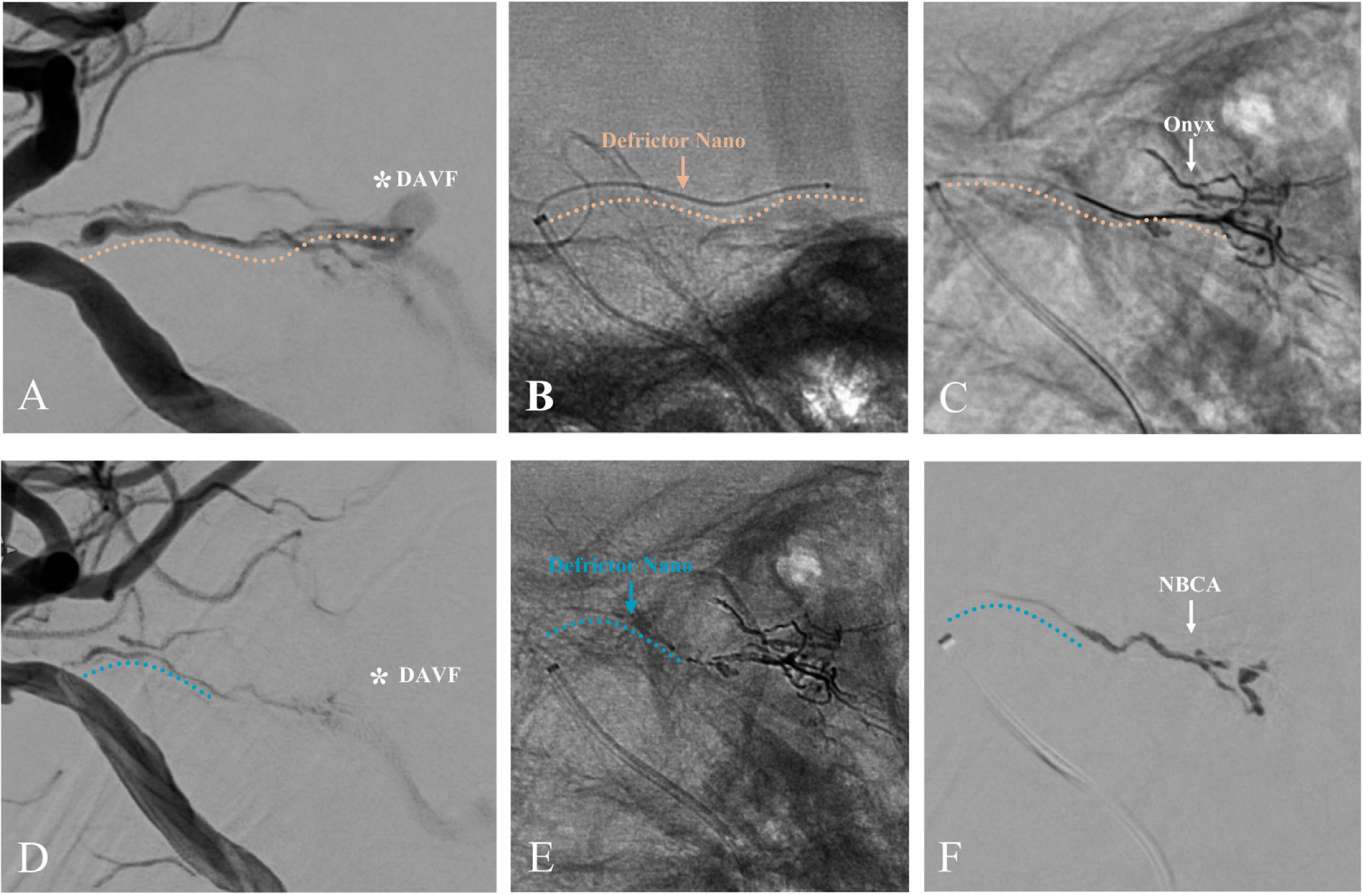
Operative images of transarterial embolization (TAE) for left tentorial dural arteriovenous fistula (TDAVF). (A,B) First embolization using Onyx: yellow line indicates the position of the microcatheter. Defrictor Nano catheter easily passes the stenotic lesion and reaches near the shunt. (C) X-ray film showing the Onyx casting. (D,E) Incomplete embolization because of the obstruction in the DeFrictor Nano. Residual DAVF is visualized (asterisk). Second embolization using n-butyl-2-cyanoacrylate (NBCA): blue line indicates the position of the microcatheter. Reapproach via the same feeder as the first embolization. We are forced to inject from the more proximal site because the area near the shunt is already filled with Onyx. Defrictor Nano reaches near the already-filled Onyx cast. (F) Superselective angiogram via a DeFrictor Nano showing that NBCA filling with the residual shunt. No significant distal dispersal or proximal regurgitation of NBCA is observed.

**Fig. 4:**
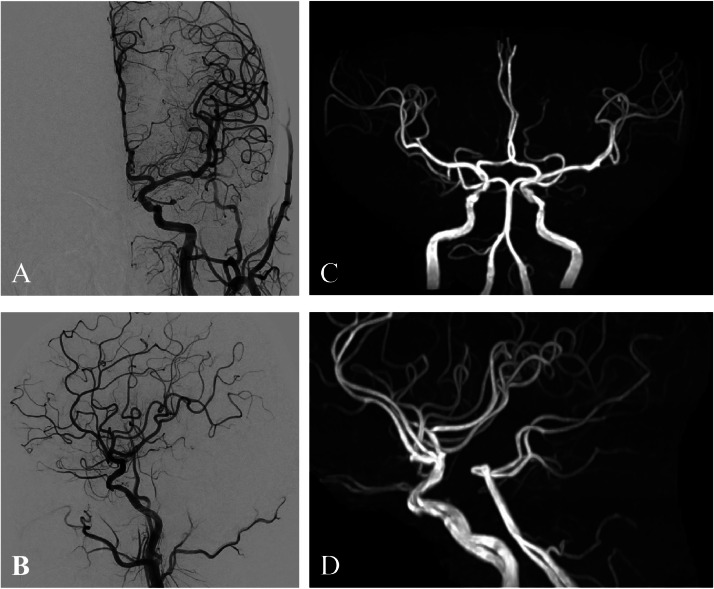
(A,B) Postoperative DSA of the left ICA showing that the DAVF has disappeared. (C,D) The disappearance of the DAVF is confirmed on follow-up MRI.

A 79-year-old man with a mild headache underwent an MRI, revealing a left tentorial DAVF (TDAVF) (Figs. 1A and B), confirmed by cerebral angiography. The TDAVF was supplied by the left tentorial, left middle meningeal, and left posterior auricular arteries, forming a shunt pouch in the left petrotentorial area. Venous drainage showed arterialisation of the left petrosal vein, creating a varix that drained into the right transverse sinus confluence. It was classified as Cognard type IV and Borden type III, indicating cerebellar cortical venous regurgitation (Figs. 1C and D). The patient opted for an endovascular approach over craniotomy. We planned TAE and determined that the left tentorial artery was the best access route due to the inaccessibility and tortuosity of the middle meningeal and posterior auricular arteries near the shunt (Figs. 1E and F). Despite stenosis at the origin of the meningeal hypophyseal trunk (MHT), the left tentorial artery provided close shunt access (Figs. 1G and H). Under general anesthesia, TAE was performed using the Onyx liquid embolic system via a transfemoral artery approach. A 9-French introducer was placed in the right femoral artery, followed by systemic heparinisation (5000 units intravenously and then 1000 units/hour infusion). A 9-French Optimo EPD guiding catheter (Tokai Medical Products, Aichi, Japan) was inserted into the right internal carotid artery (ICA), with a TACTICS PLUS intermediate catheter (Technocrat, Aichi, Japan) advanced into the cavernous segment of the ICA for stenotic passage support. The DeFrictor Nano catheter (Medico's Hirata, Osaka, Japan) and CHIKAI X 010 micro guidewire (Asahi Intecc, Aichi, Japan) navigated through the stenotic lesion to reach near the shunt (Figs. 2A-C). Onyx (0.5 mL) was injected under continuous fluoroscopy. Initially, the Onyx distribution within the shunt was optimal. However, leakage proximal to the microcatheter tip suggested a potential obstruction (Figs. 3A-C). The microcatheter was withdrawn to prevent worsening. The initial embolization resulted in incomplete occlusion, necessitating a second procedure for complete closure. If the initial attempt fails, a different vessel is typically chosen for follow-up embolization. However, in this case, alternative vessels were too tortuous to access. The partial reopening of the Onyx-embolism feeder, caused by the inadequacy of the initial procedure, exposed the shunt's access route. This suggested the possibility of successful re-embolization (Fig. 3D). We opted for a repeat embolization through the tentorial artery for total occlusion. We navigated closer to the shunt in the second embolization, staying more proximal than in the initial procedure (Fig. 3E). NBCA (0.1 mL) was injected, ensuring glue penetration at the fistula (Fig. 3F). Final left ICA angiography confirmed complete occlusion of the left tentorial DAVF (Figs. 4A and B). Follow-up MRI confirmed the disappearance of the DAVF (Figs. 4C and D). The patient was discharged without complications and lived his daily life, like before, at the 3-month follow-up visit.

## Discussion

Aggressive DAVFs, such as the TDAVF, often present with haemorrhagic symptoms due to venous hypertension from retrograde leptomeningeal drainage. Approximately 60%-75% of these cases are classified as high hemorrhage risk (Borden type III or Cognard type III/IV) [5,6]. The use of Onyx, a nonadhesive liquid embolic agent, has significantly improved TAE outcomes, facilitating controlled, prolonged injections and resulting in cure rates between 82% and 100% [1,7,8]. TDAVFs, as nonsinus type DAVFs, are known for incomplete occlusion and frequent complications from treatment [[9], [10], [11]]. Optimal arterial route selection and embolic material choice are crucial for effective and safe embolization. The middle meningeal artery (MMA) is often preferred, but its narrowness and tortuosity can limit its effectiveness [12]. In our case, we attempted TAE via the tentorial artery, traditionally seen as challenging because of its low success rates [10]. Rezende et al. reported using the tentorial artery only once in 45 TDAVFs treated with TAE (2.2%, 1/45) [13]. The tentorial artery is usually narrow and tortuous, with a limited safety margin for retrograde flow into the ICA, making it unsuitable for TAE [10,14]. In a cadaveric study, the tentorial artery predominantly originated as a single branch (80%) and is mostly the terminal branch of the MHT (90%). The mean diameter is 0.7 mm, with varying bifurcated and trifurcated origins [15]. In our case, the MHT origin was exceptionally narrow (0.25 mm), but we successfully advanced a Defrictor Nano catheter (Medico's Hirata, Osaka, Japan) with a thicker tip diameter of 0.43 mm. We hypothesize that the design of the catheter played a crucial role in this achievement. By relocating a rigid metal marker to a position 5 mm from the tip of the catheter, we ensured that the tip remained flexible, facilitating navigation without resistance and minimizing the risk of vascular injury. The structure of this microcatheter is paramount in overcoming the challenges posed by the narrowness and tortuosity of the artery. The supportability of the intermediate catheter TACTICS PLUS (Technocrat, Aichi, Japan) also played a significant role. Moreover, our approach involves strategically using different embolic materials at various stages of TAE. We initially used Onyx for its favorable properties in forming a controlled plug [1,2]. We pursued further embolization through the same feeder vessel when faced with incomplete occlusion. The goal in such cases is to place the catheter as close to the target shunt lesion as possible. However, previously placed embolic material often complicates this process, preventing close access and requiring more proximal sites for embolization. This increases the risk of regurgitation into normal vessels. NBCA is preferable in these situations due to its shorter reflux distance compared to Onyx. The low-concentration NBCA offers better visibility, allowing for precise monitoring of reflux. Additionally, NBCA's higher thrombogenicity and ability to achieve complete obliteration with a smaller volume make it an appropriate choice for further embolization [11]. The catheter wedging technique [16] and low-concentration NBCA [17] helped enhance glue penetration into the shunt. This case shows that strategic use of embolic materials, advancements in endovascular devices, and re-embolization when necessary can lead to successful outcomes, even with anatomical limitations. However, our single-center case report has limitations, and further practice in a larger number of cases is needed to validate this technique and assess the broader applicability in DAVF cases.

## Conclusions

Combining Onyx and NBCA with different liquid embolic material properties and improved catheter guidance allowed bailouts for incomplete occlusion after TAE of DAVF.

## Financial support and sponsorship

Nil.

## Patient consent

The authors certify that they have obtained all appropriate patient consent forms. In the form, the patient has given consent for their images and other clinical information to be reported in the journal. The patient understands that their name and initials will not be published and due efforts will be made to conceal their identity, but anonymity cannot be guaranteed.

## Appendix Supplementary materials

Image, application 1
